# Field evaluation of sex pheromones and binding specificity of pheromone binding protein 4 in *Tryporyza intacta* (Lepidoptera: Crambidae)

**DOI:** 10.1038/s41598-020-62092-x

**Published:** 2020-03-25

**Authors:** Yuwei Hu, Yuying Liu, Jie Bi, Ya Chen, Ya Zheng, Yongkai Mao, Yuling Mao, Hanliang Xu, Chuxiong Guan, Yan Chen, Hui Ai

**Affiliations:** 1 Guangdong Key Lab of Sugarcane Improvement & Biorefinery, Guangdong Provincial Bioengineering Institute (Guangzhou Sugarcane Industry Research Institute), Guangzhou, 510316 China; 2 Institute of Evolution and Ecology, Hubei Key Laboratory of Genetic Regulation and Integrative Biology, School of Life Sciences, Central China Normal University, Wuhan, 430079 China; 3 Engineering College, Wuhan Donghu University, Wuhan, 430212 China

**Keywords:** Behavioural ecology, Entomology

## Abstract

The recognition of chemical signal including volatile odorants and pheromones is very important in the olfactory physiological behaviors of insects, such as avoiding predators, seeking food and mating partners. The sugarcane borer, *Tryporyza intacta* is the most harmful insect in sugarcane region in Southeast Asia and Southern China, however, the study of their molecular biology and physiology was limited. Here we demonstrated that the sex pheromone (E11-16:Ald: Z11-16:Ald = 7:3) were most effective to *T. intacta*. In addition, compared the traditional rubber lure, a new microsphere formulation lure can optimize the trapping effect and might be widely used in the sugarcane growing area. To obtain a better understanding of the olfactory molecular mechanism of pheromone-based mate recognition system, we have cloned the full-length gene of the TintPBP4 and expressed in *Escherichia coli*. Our phylogenetic analysis highlighted that the TintPBP4 was highly conserved among diverse species of Lepidoptera. Furthermore, the results of QRT-PCR demonstrated that TintPBP4 transcripts were abundantly expressed in the antennae of *T. intacta*, especially in the male adults. The fluorescence binding experiments showed the TintPBP4 exhibited strong binding capacities to the sex pheromone components. These results will not only provide more understanding for the functional analysis of olfactory proteins from *T. intacta*, but also assist in the exploitation and development of sex pheromones in the integrated biological control of this pest.

## Introduction

Insects distinguish odorant molecules through their olfactory organ to seek food, mating partners and oviposition sites^1–4^. External chemicals including host-plant volatiles and sex pheromones enter into the chemosensilla and then are captured by odorant binding proteins (OBPs) in the antennae^5–7^. OBPs are water-soluble olfactory protein molecules and consist of pheromone binding proteins (PBPs) and general odorant binding proteins (GOBPs)^4,8,9^. Generally, mate finding in almost all moth lineages is dependent on males using the female-emitted pheromone, which are produced in female pheromone glands (PG) located on the abdomen terminals of a large number of lepidopteran moth species^10–12^.

In the integrated pest management, insect sex pheromones provide an environmentally friendly solution to control insect populations through mating disruption. The hydrophobic sex pheromone components that emitted by con-specifc female insects were captured and transported to pheromone receptors in the hemolymph of male antennal sensilla^13^. The first insect pheromone was identified from the silkworm moth *Bombyx mori* nearly 70 years ago and enormous progresses have been made in understanding the detection of pheromones^14^. Ma *et al*. reported male moths could be captured by trapping device with the blended baits of E10E2-16: Ald and E10E2-16:OH in *Diaphania angustalis* in the field^15^. Molnár *et al*. Found that sex pheromone E13-18:Ald could significantly attract the European pepper moth males (*Duponchelia fovealis*), and Z13-18:Ald and Z11-16:Ald also could increase the trapping effects in field experiments^16^. Binary sex pheromones (E10, E14-16: Ald and Z3, Z6, Z9-23:H) were identified from *Omphisa anastomosalis* and exhibited obvious control effect for their monitoring and suppression of field populations^17^. Similarly, Parys *et al*. indicated that pheromone lures including hexyl butyrate, (E)-2-hexenyl butyrate and (E)-4-oxo-2-hexenal (in a ratio of 4:10:7) was effective in capturing *Lygus lineolaris* through field screening^18^. The screening and functional identification of these sex pheromones are beneficial to monitoring of insect in the field.

Pheromone binding proteins (PBPs) are extracellular water-soluble proteins of around 130–150 amino acids with six or seven alpha helices and six conserved cysteines, which mainly transport sex pheromones to olfactory receptors of insect antennae^19,20^. They were first discovered from Lepidoptera and a lot of other PBP proteins were subsequently identified and characterized from the insect species of Diptera, Hymenoptera and Coleoptera^21,22^. HarmPBP1 could effectively detect to two principal sex pheromones, Z-11-tetradecenal and Z-9-hexadecenal from *Helicoverpa armigera*^23^. Similarly, Sun *et al*. revealed that the PxylPBPs not only could greatly recognize four sex pheromones of the diamondback moth (*Plutella xyllotella*) but also significantly bound their pheromone analogs^24^. Additionally, some PBP proteins could also bind host plant volatiles and play an important role in insect mating and host recognition. For instance, MvitPBP3 identified from *Maruca vitrata* showed high binding capacity with the host-plant floral volatiles, suggesting that MvitPBP3 have the similar olfactory function with the MvitGOBPs^25^. These results revealed that insect PBPs and other odorant binding proteins (OBPs) might have synergistic roles in the recognition of host-plant and mating partner.

The sugarcane borer, *Tryporyza intacta* is the most harmful pest in sugarcane region of Southeast Asia and Southern China^26^. To gain more insights into the characterization and olfactory function of pheromone binding protein 4 with sex pheromones of *T. intacta*, we first tested whether the combination of E11-16: Ald and Z11-16: Ald could attract the moth using different traps in the typical sugarcane fields. Next, the full-length gene of the PBP4 that identified from the transcriptome of *T. intacta* was cloned and expressed in *E. coli*, which mearsured their olfactory function of sex pheromone perception. Then qRT-PCR was used to test the expression pattern of *TintPBP4* genes in the different tissues of *T. intacta*. Furthermore, we determined the ligand-binding capacities of the purified TintPBP4 protein to the sex pheromone components of *T. intacta*. Present study will not only provide more understanding for the functional analysis of olfactory proteins from *T. intacta*, but also assist in the exploitation and development of sex pheromones in the integrated biological control of this pest.

## Materials and Methods

### Ethics statement

The sugarcane borer *T*. *intacta* larvae and adult moths were reared on artificial diet in the laboratory of Guangzhou Sugarcane Industry Research Institute (23°8′N, 113°17′E, Guangzhou City, Guangdong Province, China). A laboratory population was kept and maintained at 27 ± 1 °C, 70 ± 10% RH, and 14:10 h L:D. The host-plant sugarcanes (ROC22) were cultivated in the experimental field of Guangzhou Sugarcane Industry Research Institute. All experimental animal procedures including this pest were approved by the Institutional Review Board at Central China Normal University in China (CCNUIRB).

### Field experiment

In the field trapping experiment, synthetic pheromone-baited lures will trap and capture male moths inside the experimental device. Proper trap design was critical to kill the pest once it enters the trap. Different ratios of sex pheromone components (E11-16Ald: Z11-16Ald: 0/100, 100/0 and 70/30) were used to attract *T*. *intacta* in the field trials in Guangdong Province, China (20.7°N, 110.2°E). Water basin traps were used to capture sugarcane borers, which consisted of a basin and a lure hooked together by an iron wire. The iron wire passed through the basin by two holes with a lure 2 cm of the top of the water, which every trap was 50 meters apart. The traps were checked at 6 am every day and recorded the captures of *T*. *intacta*.

In addition to the traditional rubber bait, we also used a new matrix material in the trapping test, which consisting of more polymeric compound with a melting point below 100 °C. The polymer/pheromone combination was emulsified and dispersed in water. And the sex pheromones were dissolved in the matrix material together with an antioxidant. This mixture was then emulsified using a surfactant at a temperature above 80 °C. After then, these new baits were used to measure their trapping effect in the sugarcane field. *p* < 0.05 indicated significant difference by Student *t*-test.

### Cloning and sequence analysis of olfactory gene from *T. intacta*

The *TintPBP4* gene was amplified from *T. intacta* by PCR using primers, which were designed according to the gene sequence in the sequencing results of the transcriptome. PCR was performed: a denaturation step at 94 °C for 3 min; and followed by 30 cycles of 94 °C for 30 s, 56 °C for 30 s and 72 °C for 45 s and final extension for 5 min at 72 °C. PCRs were separated on a 1.0% agarose gel. Products in the estimated size range were excised, purified with a TaKaRa MiniBEST Agarose Gel DNA Extraction Kit Ver.4.0 (TaKaRa) and subcloned into pEASY-T1 vector, and positive clones were sequenced. The correct pEASY-T1-PBP plasmid was extracted as PCR template and amplified by gene specific primers with protective bases and restriction sites (BamHI and HindIII).

The *TintPBP4* cDNA sequence was analyzed using the online program BLAST (http://blast.ncbi.nlm.nih.gov/Blast.cgi) and signal peptide was predicted by Signal P 4.1 Server (http://www.cbs.dtu.dk/services/SignalP/). The amino acid sequences multiple alignments of TintPBP4 and other PBPs from *Lepidopteran* insects were analyzed and aligned using the DNAMAN software. The theoretical isoelectric point of TintPBP4 protein was obtained through the website (http://web.expasy.org/cgi-bin/protparam/protparam). The phylogenetic tree analysis of TintPBP4 with similar PBPs of other insect species were constructed by MEGA 6 software.

### Quantification of relative tissue expression

Quantitative real-time PCR test was determined by a Bio-Rad CFX 96 real-time PCR system with SYBR Green I fluorescent dye. Olfactory organ antennae and different tissues including heads (without antennae), thoraxes, abdomens, legs and wings were collected and total RNA was extracted using Total RNA kit I. cDNA was synthetized from 1 μg of total RNA using Reverse Transcriptase kit according to the manufacturer’s protocol. The mRNA transcript of TinfPBP41 was assessed by QRT-PCR with the specific primers (Table 1). The QRT-PCR reaction was performed in a final volume of 20 μl reactions containing 2 μl of cDNA (diluted 5 times from original cDNA concentration), 0.4 μl of each primer, 10 μl of TransStart Tip Green QPCR SuperMix (TransGen), and 7.2 μl of RNase-free water. The cycling conditions were as follow: 95 °C for 3 min; 40 cycles at 95 °C for 10 s and at 50 °C for 30 s; and melt curve at 65 °C to 95 °C for 5 s. Each tests included three biological replicates and three technical repetitions. The relative expression levels were calculated by the 2^−△Ct^ method, and SigmaPlot 10.0 was used to draw the histogram. *p* < 0.05 indicated significant difference by Student *t*-test.

**Table 1:** Primers used for prokaryotic expression and quantitative RT-PCR of *TintPBP4* gene.

Primer name	Sequence (5′-3′)
PBP4-BamHIF	CGCGGATCCTCGCAGGACTTGATTACGAA
PBP4-HindIIIR	CCCAAGCTTTCAAAAGTCAGTCATGATCTCC
PBP4-YF	ATCGAAGCAAGACCTCCTC
PBP4-YR	GGCTCTGTCTCGCAATCC
ActinF	TGGGTATGGAATCTTGCG
ActinR	ATCTTGATGGTGGAGGGAG

### Recombinant expression of olfactory protein

The 7.5 μl purified PCR product was ligated into cloned into 1 ul the pET-32a (+) vector by incubating the mixture with 0.5 μl T4-DNA ligase and 1 μl 10 X T4 ligase buffer at 4 °C for 16 h. The recombinant plasmid pET-32a-*TintPBP4* was transformed into *E. coli* DH5α competent cells, positive colonies were selected by their ampicillin resistance. And the constructed recombinant expression vectors were further confirmed by sequencing. Subsequently, the plasmid was transferred into *E. coli* BL21 (DE3) competent cells, and the target proteins were expressed according to a previously reported operating procedures. The crude protein fractions were further purified from the supernatant using an affinity chromatography column. The purified protein fractions were analyzed by 12% sodium dodecyl sulfate polyacrylamide gel electrophoresis (SDS-PAGE) and stored in −80 °C until use.

### Fluorescence binding assay

The binding assays were conducted following our previous studies^10^. First, we tested the binding of a fluorescent probe N-Phenyl-1-naphthylamine (1-NPN) to the protein. Next, the ligand compounds were measured in fluorescence competitive binding assays using 1-NPN as the fluorescent reporter (0.5 μM), and 0.5–10.0 μM for each competitor. Two sex pheromones from *T. intacta* were carried out in binding assays. Dissociation constants of bound ligand were calculated from the corresponding half maximal inhibitory concentration (IC_50_) values, using the equation: KD = [IC_50_]/(1 + [1-NPN]/K_1-NPN_), where 1-NPN is the free concentration of 1-NPN and K_1-NPN_ is the dissociation constant of the Protein/1-NPN complex.

## Results

### Field trapping experiment

**Figure 1:**
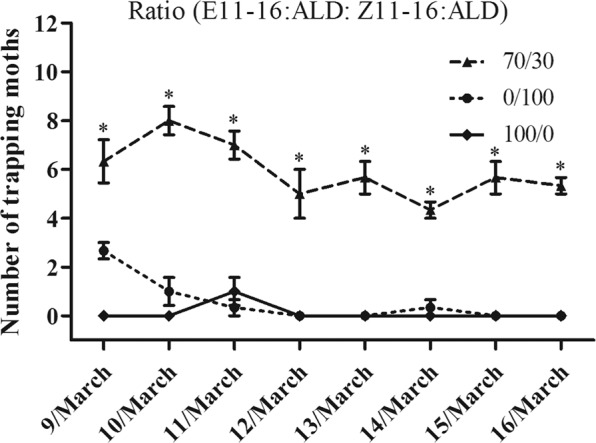
Captures of *T. intacta* per day in three water traps throughout March 9-16th of 2017 treated with sex pheromones with different ratio. *p* < 0.05 indicated significant difference by Student *t*-test.

**Figure 2:**
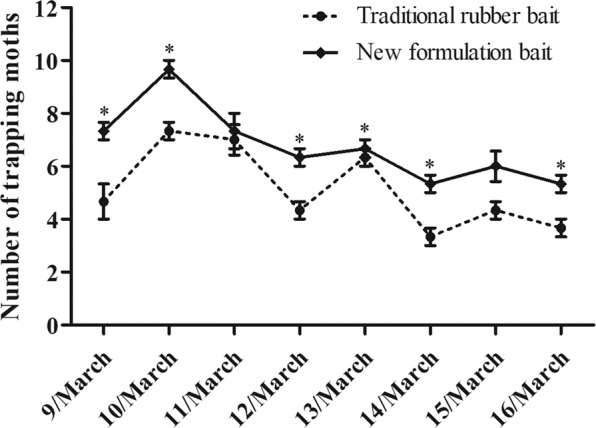
Captures of *T. intacta* per day in the field trapping experiments from March 9-16th of 2017 treated with sex pheromone with different formulations. *p* < 0.05 indicated significant difference by Student *t*-test.

The trapping experiments of different formulation baits from the sex pheromones of *T. intacta* were measured in South China. Single sex pheromone component groups (E11-16Ald: Z11-16Ald: 0/100 and 100/0) captured fewer moths in the traditional rubber bait test (Fig. 1). In contrast, the sex pheromones baits in proportion (E11-16Ald: Z11-16Ald: 70/30) catched a great number of *T. intacta* adults in the sugarcane field from March 9 to March 16 (Fig. 1). In addition, compared to the traditional rubber bait, new matrix material formulation bait with the same ratio of sex pheromones (70/30) could also effectively attract more moths of *T. intacta*. Results indicated that the number of adults trapped by the new formulation baits was significantly different from that of traditional rubber formulation (Fig. 2).

### Molecular cloning and sequence analysis of the *TintPBP4*

**Figure 3:**
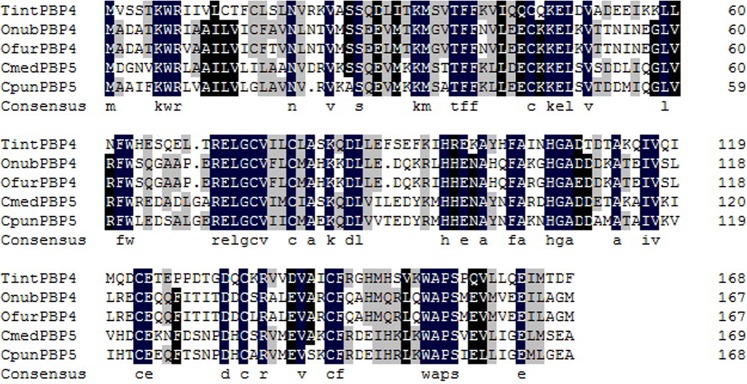
Alignment of PBPs from *Lepidopteran* insects consist of TintPBP4. Genebank number: *Ostrinia nubilalis* (ADT78493.1), *Ostrinia furnacalis* (ADT78503.1), *Cnaphalocrocis medinalis* (ALT31680.1), *Conogethes punctiferalis* (ALC76549.1).

**Figure 4:**
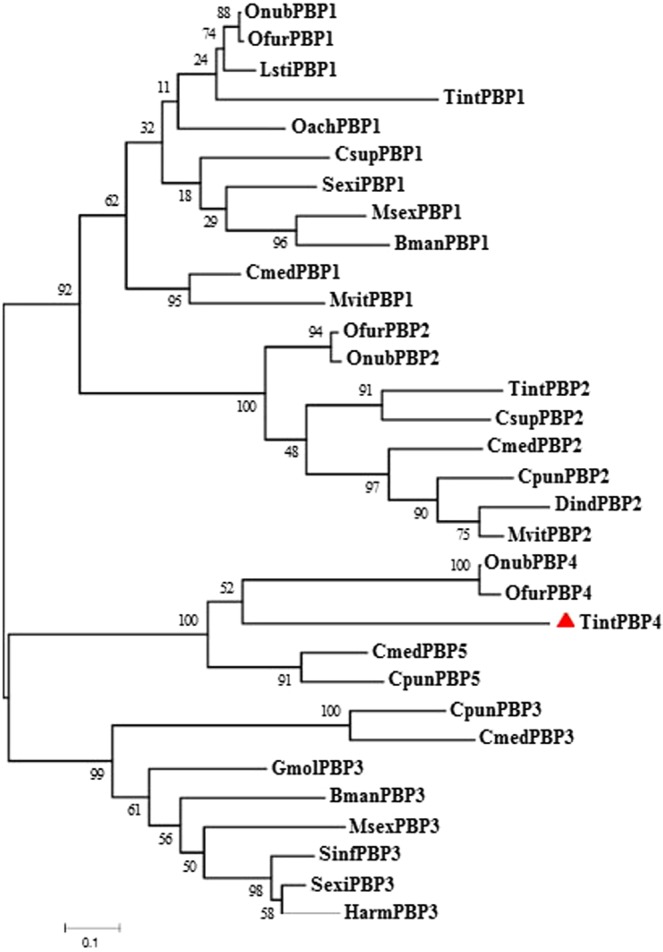
Phylogenetic tree of TintPBPs amino acid sequence with other PBPs from different insect species. The tree was constructed by the neighbor-joining method of MEGA. GenBank accession numbers: OnubPBP1 (ADT78495.1), OfurPBP1 (ADT78500.1), LstiPBP1 (ACD67881.1), TintPBP1 (MF624766), OachPBP1 (AEZ52490.1), CsupPBP1 (ACJ07123.1), SexiPBP1 (AAF06123.1), MsexPBP1 (AAA29326.1), BmanPBP1 (ACT34881.1), CmedPBP1 (AFG72997.1), MvitPBP1 (AGS46557.1), TintPBP2 (MF624767), CsupPBP2 (ADK66921.1), CmedPBP2(AGI37364.1), CpunPBP2 (ALC76550.1), DindPBP2 (BAG71419.1), MvitPBP2 (AGS46555.1), CpunPBP3 (ALC76551.1), GmolPBP3 (AHZ89399.1), BmanPBP3 (ACW84370.1), MsexPBP3 (AAF16702.1), SinfPBP3 (AEQ30020.1), SexiPBP3 (ACY78413.1), HarmPBP3 (AAO16091.1), OnubPBP4 (ADT78493.1), OfurPBP4 (ADT78503.1), CmedPBP5 (ALT31680.1), CpunPBP5 (ALC76549.1).

To characterize the *TintPBP4* gene from *T. intacta*, a 507 bp open reading frame (ORF) was first amplified, cloned, and sequenced. The *TintPBP4* ORF encodes 169 amino acids and contains a putative hydrophobic signal peptide with 26 amino acids at the N-terminus. The predicted molecular weight of PBP is 16.39 kDa and the theoretical isoelectric point is 5.13. The amino acid multiple alignment result indicated that TintPBP4 shared 42.35%, 42.35%, 48.82% and 43.53% sequence identity with OnubPBP4, OfurPBP4, CmedPBP5 and CpunPBP5, respectively (Fig. 3). The phylogenetic analysis showed that PBPs of *Lepidopteran* were significantly divided into different groups, PBP1 and PBP2 are clustered while PBP3, PBP4 and PBP5 are clustered, respectively (Fig. 4).

### Tissue and sex dependent distributions of *T. intacta PBP4*

**Figure 5:**
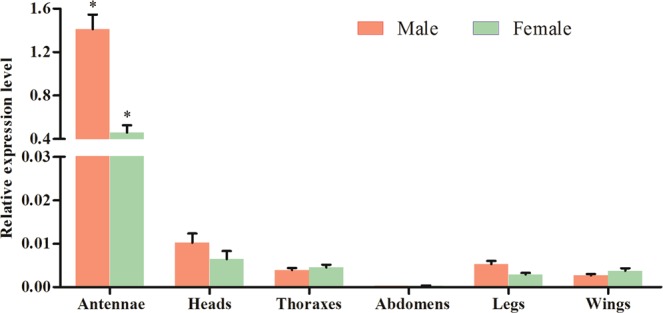
Relative transcript levels of *TintPBP4* in different adult tissues measured by qRT-PCR. *p* < 0.05 indicated significant difference by Student *t*-test.

We used qRT-PCR to assess the transcript abundance of *TintPBP4* genes and that showed varying degrees of expression in different tissues. Our result indicated that *TintPBP4* transcript was highly expressed in antennae, and low expression in head, thoraxes, abdomen, legs and wings (Fig. 5). Moreover, the *TintPBP4* gene distribution was sex-biased and specially expressed in male antennae with 3.08-fold difference compared to that of female moths.

### Expression and purification of recombinant protein

**Figure 6:**
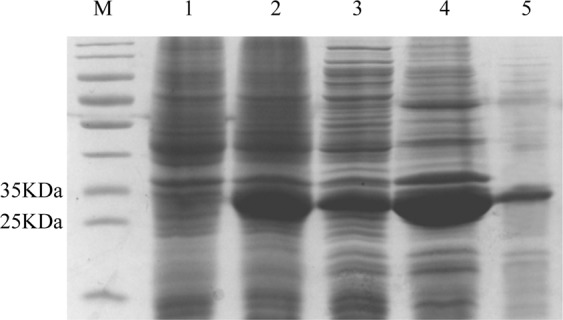
Sodium dodecyl sulfate polyacrylamide gel electrophoresis (SDS-PAGE) analysis of recombinant TintPBP4. Lane M - marker protein, Lane 1 - noninduced *Escherichia coli* TintPBP4, Lane 2 - induced *E. coli* TintPBP4, Lane 3 - supernatant after broken, Lane 4 - precipitation after broken; Lane 5 - purified TintPBP4.

The *TintPBP4* gene was abundantly expressed in *E. coli* after IPTG induction. The crude protein fraction was purified using an affinity chromatography column (Huijin pro Ni-4FF (IDA)), along with the manufacture’s protocols. The purified protein fractions were analyzed by SDS-PAGE (Fig. 6). Recombinant PBP4 protein was stored at −80 °C until used in the binding experiment.

### Fluorescence binding capacity of TintPBP4

**Figure 7:**
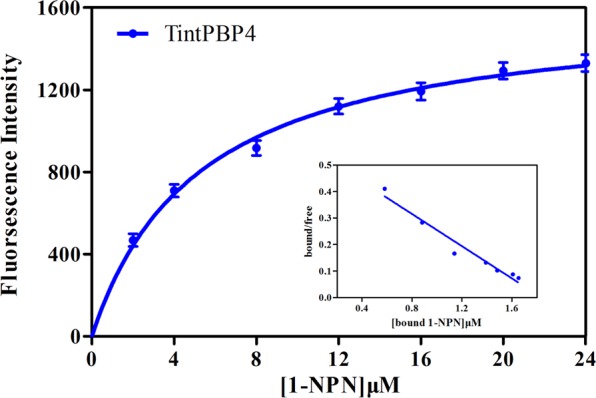
Binding curve of N-phenyl-1-naphthylamine (1-NPN) to recombinant TintPBP4.

**Figure 8:**
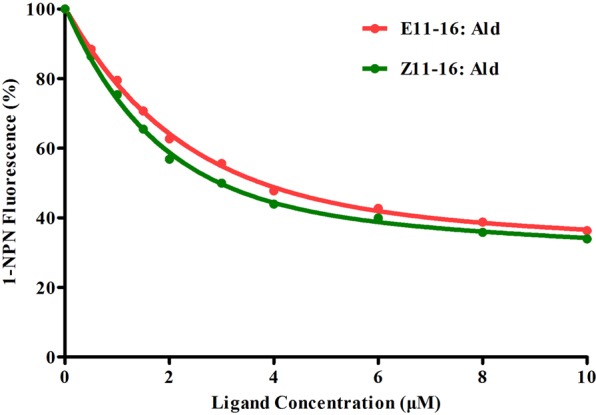
Competitive binding curves of selected ligands to the protein TintPBP4.

The fluorescence binding assay was performed by measuring the affinity of TintPBP4 to a fluorescent probe N-Phenyl-1-naphthylamine (1-NPN). An increase in the fluorescence intensity at 337 nm was observed when increasing amounts of 1-NPN were added to the TintPBP4. The binding curves and Scatchard plots demonstrated that the binding of fluorescent ligand and TintPBP4 increases with increasing concentrations of the 1-NPN (Fig. 7). The PBP4 protein had high binding affinities with the principal sex pheromone component E11-16: Ald and Z11-16: Ald of *T. intacta* and IC_50_ values were 3.86, 3.29, respectively (Fig. 8 and Table 2). These results showed that this PBP protein had binding *T. intacta* sex pheromones and play a vital role in the olfactory signal transduction in adult moth mating.

**Table 2:** The binding constants of different ligands. Binding of 1-NPN and different sex pheromone components to TintPBP4.

No.	Compounds	TintPBP4	
		IC_50_ (μM)	*K*_i_ (μM)
1	E11-16: Ald	3.86	2.97
2	Z11-16: Ald	3.29	2.51

Note: IC_50_, ligand concentration displacing 50% of the fluorescence intensity of the TintPBP/N-phenyl-1-naphthylamine complex; *Ki*, dissociation constant.

## Discussion

Insects can recognize a variety of volatile compounds from pheromone glands and host plant that stimulate specific behaviors, such as mating, feeding and egg laying by chemoreceptive organs^23,24^. Pheromone molecules and food odors are detected by a fine chemosensory system, whose olfactory neurons mainly reside in the antennae and maxillary palps^27^. Pheromone-binding proteins (PBPs), a subfamily of odorant-binding proteins, are thought primarily to bind and transport the sex pheromones in moths^25,26^. Since *T. intacta* is a serious pantropical pest, according to the integrated pest management (IPM) idea, people used its sex pheromone to control it in the sugarcane production of southern China^27,28^. However, there are also some problems in the stability and persistence when the traditional rubber bait of sex pheromones from *T. intacta* was used in the field. Therefore, we need to design and use new material or formulation to improve the preventive control effect of their sex pheromones in the trapping test.

The sex pheromone of *T. intacta* females attracted male moths using a blend of sex pheromones as different proportion. Our field trapping result revealed that two sex pheromone components, E11-16: Ald and Z11-16: Ald could effectively attract more male *T. intacta* in a ratio of 70: 30. When a single sex pheromone component was used alone, fewer adults are trapped in March 9-16. The overwintering period of *T. intacta* mainly harms the sugarcane seedlings in March, while the next generation pests mainly harm the stems of sugarcane in July and August. In our previous study, we also confirmed both of sex pheromone components (70: 30) of *T. intacta* could effectively attract a great number of adult moths in July^10^. In addition, new formulation bait was used to trap moths and traditional rubber bait was also evaluated as the comparison. This new formulation bait with matrix material showed obvious enhancement effect and more moths were captured in the field test. Experimental results demonstrated that the new formulation could optimize the trapping effect and may be widely used in trapping experiments and population monitoring in the sugarcane field.

The expression levels in the heads, thoraxes, abdomens, legs and wings of *T. intacta* were significantly lower than that of antennae, suggesting their functional similarity with other PBPs of Lepidoptera species. Furthermore, QRT-PCR demonstrated that the expression patterns of *TintPBP4* gene exhibited obvious male-biased (3.08-fold difference) in the antennae of *T. intacta*. Our previous study showed that the expression level of *TintPBP1-2* genes in the antennae of male moths was significantly higher than the female adults, while the expression of *TintPBP3* gene in the female antennae of *T. intacta* was more abundantly expressed than the male moths^10^. Form these findings, the male-biased expression of the *TintPBP4* and *TintPBP1-2* genes indicated that they may play an essential role in the recognition of male moths to sex pheromones released by the pheromone gland of female *T. intacta*. Additionally, we found TintPBP4 was very sensitive to E11-16: Ald and Z11-16: Ald, which its *Ki* value was 2.97 μM and 2.51 μM, respectively. This strong binding capacity was also revealed in both TintPBP1 and TintPBP2 in our previous research^10^. These results also revealed that the TintPBP1-2 and TintPBP4 proteins play the main roles in the sexual communication and mating of *T. intacta*.

Male moths locate their mates using species-specific sex pheromones emitted by conspecific females. Pheromone binding proteins (PBPs) are supposed to contribute to the sensitivity and selectivity of pheromone detection in moths. In summary, we showed that TintPBP4 protein could contribute to the sensitivity of pheromone detection and olfactory functional analysis. Our results could expand knowledge of the pheromone trapping formulation in *T. intacta*, which will help to provide novel technologies to monitor and control this sugarcane borer in the field.
